# Impacted maxillary canines and root resorption of adjacent teeth: A retrospective observational study

**DOI:** 10.4317/medoral.21337

**Published:** 2016-10-01

**Authors:** Rosanna Guarnieri, Costanza Cavallini, Roberto Vernucci, Maurizio Vichi, Rosalia Leonardi, Ersilia Barbato

**Affiliations:** 1 PhD Student, Department of Maxillofacial Sciences, School of Dentistry, “Sapienza” University of Rome, Rome, Italy; 2 Research Assistant, Department of Radiological Sciences, Oncology and Pathological Anatomy, “Sapienza” University of Rome, Rome, Italy; 3 Professor, Department of Statistics, “Sapienza” University of Rome, Rome, Italy; 4 Professor, Department of Orthodontics, University of Catania, Catania, Italy; 5 Professor, Department of Maxillofacial Sciences, School of Dentistry, “Sapienza” University of Rome, Rome, Italy

## Abstract

**Background:**

The prevalence of impacted maxillary canine is reported to be between 1% and 3%. The lack of monitoring and the delay in the treatment of the impacted canine can cause different complications such as: displacement of adjacent teeth, loss of vitality of neighbouring teeth, shortening of the dental arch, follicular cysts, canine ankylosis, recurrent infections, recurrent pain, internal resorption of the canine and the adjacent teeth, external resorption of the canine and the adjacent teeth, combination of these factors. An appropriate diagnosis, accurate predictive analysis and early intervention are likely to prevent such undesirable effects. The objective is to evaluate, by means of a retrospective observational study, the possibility of carrying out a predictive analysis of root resorption adjacent to the impacted canines by means of orthopantomographs, so as to limit the prescription of additional 3D radiography.

**Material and Methods:**

120 subjects with unilateral or bilateral maxillary impacted canine were examined and 50 patients with 69 impacted maxillary canine (22 male, 28 female; mean age: 11.7 years) satisfied the inclusion criteria of the study. These patients were subjected to a basic clinical and radiographic investigation (orthopantomographs and computerized tomography). All panoramic films were viewed under standardized conditions for the evaluation of two main variables: maxillary canine angulations (a, b, g angles) and the overlapping between the impacted teeth and the lateral incisor (Analysis of Lindauer). Binary logistic regression was used to estimate the likelihood of resorbed lateral incisors depending on sector location and angle measurements.

**Results:**

Results indicated that b angle has the greatest influence on the prediction of root resorption (predictive value of b angle = 76%). If β angle <18° and Lindauer = I, the probability of resorption is 0.06.

**Conclusions:**

Evaluation of b angle and superimposition lateral incisor/impacted canine analysed on orthopantomographs could be one of the evaluation criteria for prescribing second level examination (CT and CTCB) and for detecting root resorption of impacted maxillary canine adjacent teeth.

**Key words:**Impacted canine, root resorption, panoramic radiography, angulation, prediction.

## Introduction

The prevalence of impacted maxillary canine is reported to be between 1% and 3% (1-3).

The lack of monitoring and the delay in the treatment of the impacted canine can cause different complications such as (4): displacement of adjacent teeth, loss of vitality of neighbouring teeth, shortening of the dental arch, follicular cysts, canine ankylosis, recurrent infections, recurrent pain, internal resorption of the canine and the adjacent teeth, external resorption of the canine and the adjacent teeth, combination of these factors.

An appropriate diagnosis, accurate predictive analysis and early intervention are likely to prevent such undesirable effects.

Ericson and Kurol (1987a; 1987b) (5,6), in a study led with the aid of plane film radiography, showed an incidence of central and lateral incisors root resorption, due to impacted maxillary canines, of 12.5% of the sample.

However, 2D radiographs may lead to an underestimation of the problem, since root resorption becomes clearly visible only when the entire thickness of the root surface, from the lingual to the buccal surface, is damaged, or when it is sufficiently advanced so as to alter the mesio-distal profile of the root. Resorption is also difficult to diagnose on orthopantomograms due to overlapping of the incisor and the canine, and because the degree of resorption should be compared with the initial thickness of the root (6).

Ericson and Kurol (2000) used computerized tomography to investigate the position of maxillary canines and the prevalence of incisor root resorption (7). In this study, CT revealed signs of root resorption in almost half of the sample (38% of the lateral and 9% of the central incisor), demonstrating that CT allows a more precise diagnosis of the phenomenon than conventional radiography. Also, CT facilitates observations of even minor loss of dentine on the roots of teeth (8,9) .

Walker *et al.* (2005) have carried out a study with the aim of evaluating the spatial relationships of the impacted canines relative to adjacent structures and incisor resorption with cone-beam computed tomography (10). A strong correlation was found between resorption of the incisors and impacted canine, affecting 66.7% of lateral incisors and 11.1 % of central incisor.

Similar results (Haney 2010) had led the Authors to the conclusion that 2D and 3D images of impacted maxillary canines can produce different diagnoses and treatment plans, due to lack of congruence (36%) in the perception of root resorption of adjacent teeth (11).

- Root resorption and gender.

Incisor resorption is more common in female than in male, with a female/male ratio varying between 2:1 (8,11-13), 3:1 (14), 4:1 (6,10,15) and the highest ratio being 10:1 (16). On the contrary, a recent study found no significant relationship between the occurrence of resorption and gender (17).

- Root resorption and type of inclusion.

Resorption was usually unilateral, a further indication that also local factors can be responsible (6).

- Root resorption and site of inclusion.

The position of the impacted canines has an effect on the process of resorption of incisor roots. A correlation was found between the proximity of the impacted canine to the incisors and their resorption (10). The distribution of the resorption on the roots of the incisors was in agreement with the positions of the crown of the ectopic canines. Physical proximity (\1 mm) between the impacted canine and an adjacent root is the most important predictor for root resorption (18).

- Teeth resorbed

The most affected tooth is always the lateral incisor (80.5% to 85.5%) (7,6) followed by central incisor (9% to 12.7%) (7,6). Resorption can potentially affect at the same time all upper incisors, although it is very improbable (16). Bicuspid root resorption must be considered very unusual (2.12%) (9).

- Root resorption and contact relationship between the canine and the lateral incisor.

Since 1987, Ericson’s study confirmed that the contact point between the cuspid and the neighbouring tooth demonstrated to be the site of resorption in all instances. These results suggest that pressure may be a trigger factor, even though there may be other factors involved in the aetiology of resorption. Close contact between the canine and the lateral incisor can be the cause of the resorption, rather than the swelling of the dental follicle and even in the absence of systemic factors (16,19). In particular, the high pressure deriving from the contact between the canine crown and the root leads to the activation of resorptive processes (17).

- Root resorption and overlapping lateral incisor/canine (panoramic radiography).

Superimposition of the incisor roots and the crown of impacted canine on intraoral radiographs and on panoramic radiography obscured the root anatomy in 45% of cases (5). Resorption was consistently found in patients having the cusp of the maxillary canine positioned mesially to the midline of lateral incisor in panoramic and periapical films. Therefore, a typical candidate for resorption of the lateral incisors during ectopic eruption of the maxillary canine is a patient with a well-developed canine root, erupting medially to the long axis of adjacent incisor (7).

- Location of resorption

A conventional radiographic study (Ericson and Kurol 1988) has shown that resorption was more common in the middle third of the root (6,14). Therefore, apical and middle third involvement was the most common pattern, as confirmed by subsequent CT studies of Ericson and Kurol (2000) (7) and Rimes (1997) (12). Cernochova’s study (2011) (17) showed that the most severe root resorption occurred in the apical third (57.6%).

- Degree of resorption

Ericson and Kurol (2000) in a CT study (7) observed that 60% of the resorption of the lateral incisor and 43% of the central incisors had pulpal involvement. Most of the root resorption cases were advanced and without clinical signs or symptoms when diagnosed.

Also Rimes (1997) had previously found that resorption tended to be extensive: 30 out of 35 resorbed teeth had pulpal involvement (12).

- Root resorption and angular measurements.

Ericson and Kurol (1988) (14) found that the risk of resorption increases by 50% when the eruption inclination (angle A, canine inclination to the midline) exceeds 25 degrees, and increases by 50% when the eruption inclination (angle B, canine inclination to the axis of the lateral incisor) exceeds 28 degrees, as compared with the controls.

The same Authors (2000) confirm that resorption of the lateral incisors during ectopic eruption of the maxillary canine is more probable when the tooth erupts medially to the long axis of adjacent incisor, and it is inclined at an angle of 25 degrees or more to the midline of the jaw (7).

## Material and Methods

In this study an analysis of the pre-treatment records of 1674 Caucasian patients treated at Department of Orthodontics of “Sapienza” University of Rome and at the Department of Orthodontics of the University of Catania, Italy, was performed following the approval of the regional Ethical Review Board of the “Umberto I” General Hospital of Rome (Rif. 3755).

120 subjects with maxillary impacted canine were examined. Of 120 selected patients, 50 patients (22 male, 28 female; mean age: 11,7 years) satisfied the inclusion criteria of the study, namely: no previous orthodontic treatment; no subject affected by craniofacial syndromes, nor sequelae of traumatic injuries; earliest available pre-treatment panoramic radiographs realized in mixed or permanent dentition; CT imaging of the upper alveolar bones obtained after panoramic radiographs in order to evaluate the impacted canine; good quality radiograph views of canine regions available for each patient.

Panoramic radiographs were taken with a Planmeca 2002CC Proline Machine with a peak voltage of 68 KWp and current of 7 mA and an Exposure Time of 18 s.

A Siemens Somatome Plus CT-scanner (Siemens AG, Germany) was used for the tomographies with a peak voltage of 120 KWp and current of 90 mA and an Exposure Time of 3 s.

The initial phase of the data collection process involved the elaboration of an excel worksheet for data categorization against the following 20 variables ( Table 1).

**Table 1:**
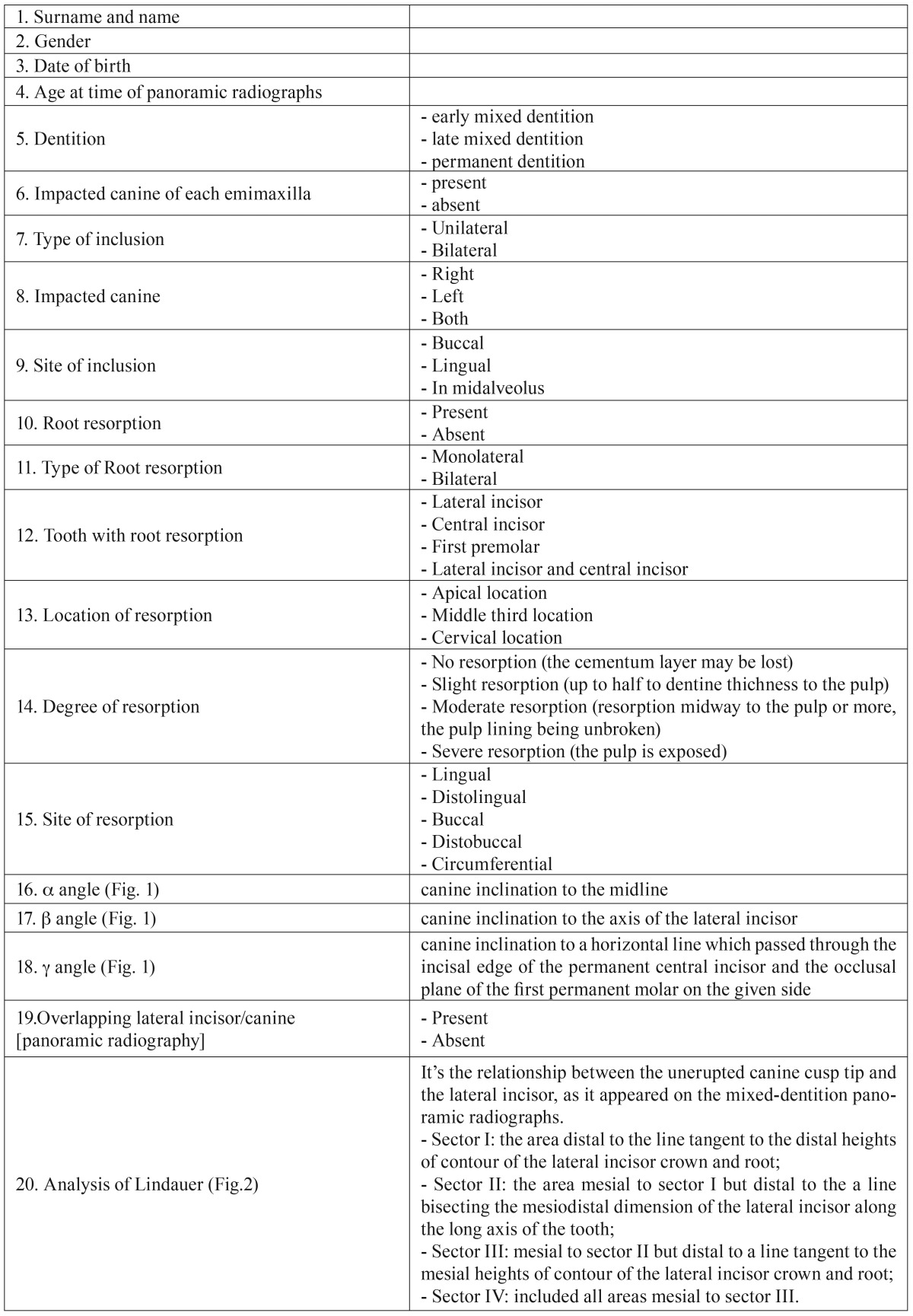
Data collection.

**Figure 1:**
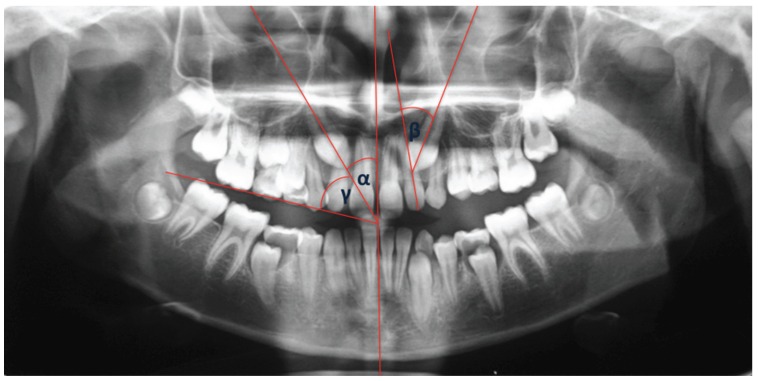
α, β, γ angle.

**Figure 2:**
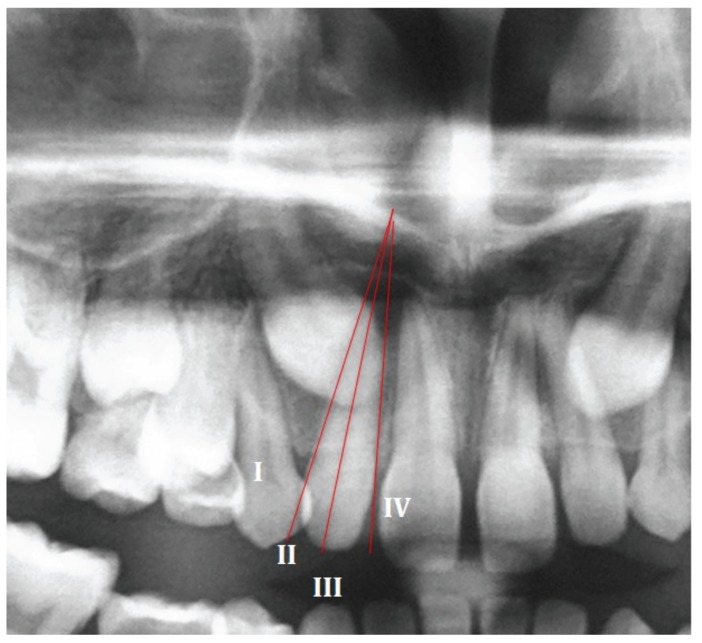
Analysis of Lindauer.

All pretreatment panoramic films were viewed under standardized conditions and traced onto acetate tracing paper with a 0.2 mm-diameter lead pencil by the same two operators (R.G. and C.C.) for the evaluation of two main variables: maxillary canine angulations by α angle, β angle, γ angle (Fig. 1,  Table 1), and the analysis of Lindauer (Fig. 2).

The evaluation of the tooth resorption was made considering the contiguous transverse CT scans (from 6 to 10, obtained from the cervical region to the apical region), with a thickness of 2 mm, perpendicular to the long axis of the lateral.

- Statistical Analysis

The investigator underwent an intra-examiner reliability check for both angulation and sector. Ten randomly selected panoramic radiographs were measured twice according to procedures mentioned, with 14 days separating the measurement sessions. Correlations (Pearson r or Spearman ρ) between measurements on these occasions were 0.995 for angulation and 1.000 for sector [*P* < 0.0001].

Relationships of data were studied by the chi-squared test. Binary logistic regression was used to estimate the likelihood of resorbed lateral incisors depending on sector location and angle measurements.

## Results

The sample consisted of 50 patients (69 impacted maxillary canine), including 22 male subjects (44%) and 28 female subjects (56%), aged between 9 and 15 years (mean age: 11.7 years). The sample had the following distribution by dentition: early mixed dentition: 10% (5), late mixed dentition 42% (20) and permanent dentition: 48% [24]. 100 maxillary canines were evaluated, of which 69% [69] impacted, and the remaining 31% [31] with a physiological eruption; of the 50 subjects under examination, 31 (62%) had unilateral impaction, while 19 (38%) had bilateral impaction. The 36% of the sample had an inclusion of the right maxillary canine, while 26% showed an inclusion of the left maxillary canine, and the remaining 38% belonged to the group of bilateral inclusions. The most frequent site of inclusion was the palatal (72%, 50), followed by the buccal (19%, 13), and inclusions in midalveolus (9%, 6).

The analysis performed showed that the resorption, measured on CT, was 24% [24] of the total sample and 34% of the sample of impacted canine. Only 1 resorption was bilateral (4%), against 22 cases of unilateral resorption (96%). In 92% (20) of the case of resorption, lateral incisors were resorbed, while central incisors were resorbed in 4% (1) of cases, and in the remaining 4% (1) both central and lateral incisors were resorbed. Apical third is the pattern of resorption in 52% (12) of cases, while 48% (11) of the resorption was located in the middle third. 70% (16) of the resorption was slight, 26% (6) moderate, while 4% (1) of affected teeth had a severe resorption. 74% (17) of resorption was observed on the palatal side of the root of the lateral/central incisor, 26% (6) on the buccal side. It is also possible to make a further distinction between purely palatal resorption (35%, 8) and distopalatal resorption (39%, 9), and between purely buccal resorption (17%, 4) and distobuccal (9%, 2).

A significant correlation was found between root resorption and inclusion (*p* value=0.002). Statistical correlation was found between the site of root resorption and the site of inclusion (*p* value=0.002). The only circumferential resorption was found in a lateral incisor close to a normally erupting canine, while midalveolus inclusions were the only kind of inclusions that did not cause resorption. There were no statistically significant sex differences in the analysed factor.

The overlapping between lateral incisor and maxillary canine viewable on panoramic radiographs was present in 54% [54] of cases, while it was absent in 46% [46] of cases. In case of resorbed teeth adjacent to impacted canine, superimposition between lateral incisor and maxillary canine was present in 91% (20) of cases. In the assessment of Lindauer’s sector analysis impacted maxillary canine was located in sector I in 22% (15) of cases, in sector II in 22% (15) of cases, in sector III in 19% (13) of cases and in sector IV in 37% [26] of cases.

Different mean values ​​were observed in the evaluation of angular measurements in relation to the presence or absence of root resorption. The mean values ​​of the angles a, b, g are 30°, 42°, 50° for the resorbed incisors, and 21°, 28°, 61° for the not resorbed incisors, respectively.

Predictability of root resorption as a function of sector location and angulation was estimated through binary logistic regression. Results indicated that b angle has the greatest influence on the prediction of root resorption ( Table 2). The predictive value of b angle is equal to 76%, while a and g angles do not seem to be statistically significant. Therefore, the probability of root resorption was calculated based exclusively on the most appropriate variables, namely b angle and Sector Angulation. The values of b angle were divided in 4 equal ranges of about 18° each, based on the maximum and minimum angles found in the data. Binary logistic regression shows that ( Table 3):

**Table 2:**
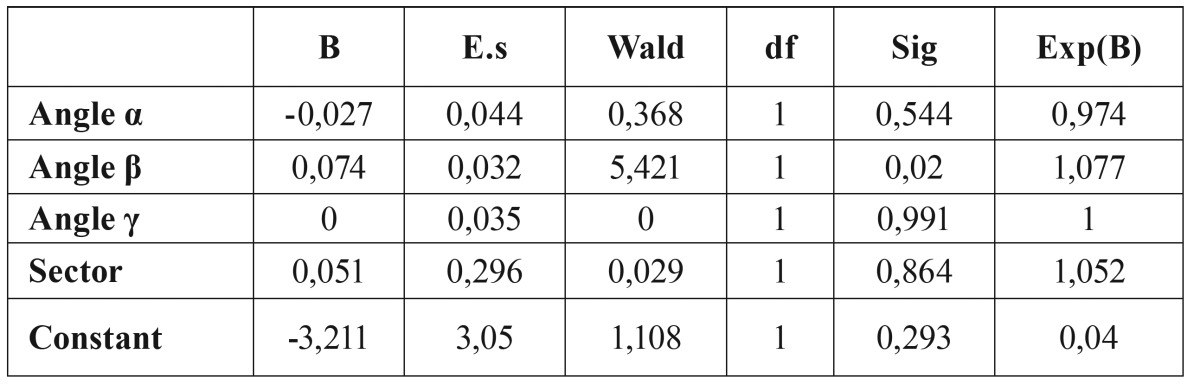
Probabilities of root resorption of the various combination of angulation and Sector.

**Table 3:**
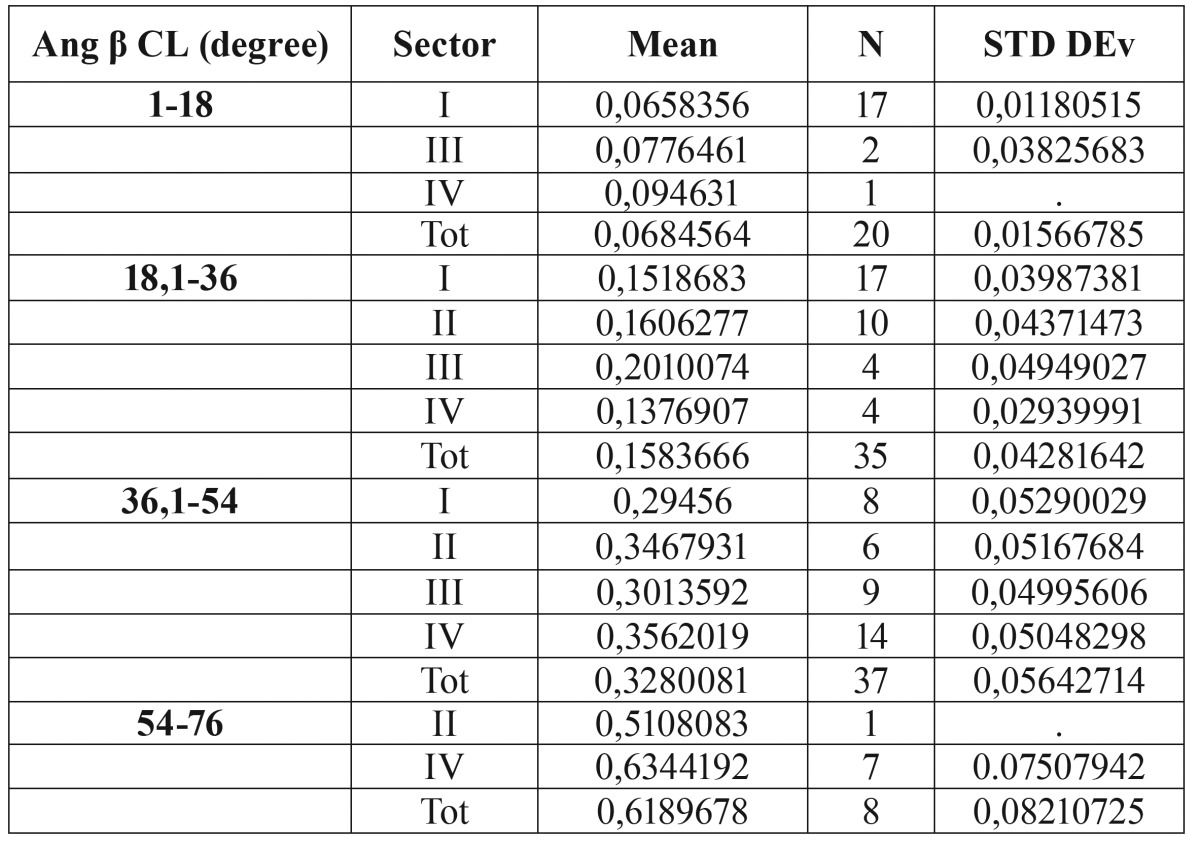
Binary logistic regression.

• If β angle > 54°, the probability of having root resorption is greater than 61%, regardless of the evaluation of the analysis of Lindauer.

• If β angle <18° and Lindauer = I, the probability of resorption is 0.06%.

## Discussion

In this CT study, resorption on the roots of the incisors adjacent to the ectopically positioned canine occurred in 34% of cases. This percentage is lower than that found by Ericson and Kurol in the similar CT study (47%) (7), but it is very similar to that found by the recent study of Alquerban A. (33.8%) (20). However, the highly significant correlation between root resorption and the presence of inclusion (*p* value=0.002), in agreement with the literature, demonstrates a real risk of resorption in the case of inclusion of the maxillary canine. The 74% of resorption was observed on the palatal side of the root. Since canine ectopic lingual eruption is much more common than buccal eruption, resorption is more frequent on the lingual side of the root. Moreover, distopalatal resorption is more common than purely palatal, probably due to the eruptive path of the canine.

Apical third is the most common pattern of resorption (52%) in contrast with other studies (6,14). Despite the high percentage of resorptions, fortunately the 70% of these was slight, with no pulp involvement.

The fact that resorption in this sample is mainly unilateral (only one resorption is bilateral) is an indication of the central role played by local factors (6), as confirmed by the significant correlation between the site of inclusion and the resorption (X2= 0,002). Palatal root resorptions are associated with palatal impacted teeth, and buccal root resorptions are associated with buccal canines. The correlation is even stronger when considering the distal/mesial tilt of the root. Furthermore, it was observed that midalveolus inclusions (which have a low probability of crown-root contact point) are the only type of inclusions that did not cause resorption.

There is no scientific evidence to support the use of CBCT as the first survey in the evaluation of the inclusion of the maxillary canine. CBCT should be performed only when the traditional radiography is unable to provide the adequate information. The panoramic radiograph, a routine examination in dental practice, is a valuable for assessing the need for second-level exams (CT and CBCT).

## Conclusions

• Strong correlation exists between inclusion and resorption. The preventive approach, consisting of early diagnosis of ectopic eruption of maxillary permanent canines, is the gold standard for the treatment of root resorption.

• β angle is more significant than a and g angles and has greater predictive power than the analysis of Lindauer. The probability of resorption is higher than 61% if the β angle>54°. In addition, the probability of resorption is very low (0.06%) if the canine is located in Sector I of Lindauer and the β angle is less than 18°.

• The prescription of CT or CBCT in the case of inclusion of the maxillary canine and evaluation of the resorption of the adjacent teeth is strongly recommended when preliminary evaluation of panoramic radiograph shows that:

- There is overlapping between the lateral incisor and the canine;

- Lindauer’s sectorial analysis is positive for the sector II, III, IV;

- The β angle is > 54°.
